# Draft genome sequence of *Brevibacillus borstelensis* strain BB2 isolated from a hot spring in the Cochamó Valley, Chile

**DOI:** 10.1128/mra.00454-26

**Published:** 2026-09-11

**Authors:** Alex Gonzalez, Nancy Calisto-Ulloa, Natalia Valdés, Bárbara Seguel-Vinés, Carlos Aranda, Aparna Banerjee, Francisco Issotta

**Affiliations:** 1 Programa de Investigación en Biomedicina y Biotecnología, Departamento de Salud, Universidad de Los Lagos 28061, Osorno, Chile; 2 Química y Farmacia, Departamento de Salud, Universidad de Los Lagos 28061, Osorno, Chile; 3 Biomedical Engineering, Faculty of Engineering, Universidad de Santiago de Chile 120774 , Santiago, Chile; 4 Departamento de Ciencias Biológicas y Biodiversidad, Universidad de Los Lagos 28061, Osorno, Chile; 5 Functional Polysaccharides Research Group, Instituto de Ciencias Aplicadas, Facultad de Ingeniería, Universidad Autónoma de Chile 125381, Talca, Chile; 6 Centro Científico y Tecnológico de Excelencia Ciencia & Vida, Santiago, Chile; 7 Facultad de Ciencias, Universidad San Sebastián 28088, Santiago, Chile; University of Rhode Island, Kingston, Rhode Island, USA

**Keywords:** *Brevibacillus borstelensis*, thermophilic bacterium, environmental microbiology

## Abstract

*Brevibacillus borstelensis* strain BB2 was isolated from a soil sample collected in the Cochamó Valley, Chile. The draft genome comprises 187 contigs totaling 5.25 Mb with a guanine–cytosine (GC) content of 51.7%. Sequencing on an MGI DNBSeq-G400 platform generated 3,406,660 paired-end reads, providing approximately 170× coverage for genomic resource reporting.

## ANNOUNCEMENT

*Brevibacillus borstelensis* is a thermophilic, spore-forming, gram-positive bacterium commonly isolated from soil and extreme environments, such as hot springs (1, 2). Strain BB2 was obtained from a soil sample collected at the Ralún hot spring in the Cochamó Valley, Chile (41°21′40.3″S, 72°19′48.4″W; WGS84), an area influenced by volcanic activity. Members of *B. borstelensis* from geothermal environments are of biotechnological interest, with prior work characterizing thermal stress responses in this species (1, 3, 4), yet genomic resources remain scarce. Sequencing strain BB2 addresses this gap and provides a basis for identifying genes underlying its adaptation to its hot spring habitat. Genome annotation revealed the presence of genes encoding esterases/lipases, siderophore-associated functions, stress response mechanisms, and sporulation-related systems, providing genomic information relevant to the environmental persistence and physiological traits of this strain (3, 4).

The soil sample was collected at a depth of 10 cm using sterile tools and stored at 4°C until processing. To isolate viable bacterial strains, aliquots were suspended in sterile NaCl 0.9% and serially diluted before plating on LB/3 agar (in g/L: yeast extract 1.7; NaCl 3.3; peptone 3.3; agar 20) and incubating at 50°C for 24 h. Colonies with Brevibacillus-like morphology were purified by repeated streaking and cryopreserved at −80°C in 20% glycerol. A single colony was cultured in LB/3 agar at the same conditions mentioned above for genomic DNA extraction. Total genomic DNA was extracted using the GeneJET Genomic DNA Purification Kit (Thermo Fisher) according to the manufacturer’s instructions. The DNA was quantified fluorometrically on a Qubit 4.0 fluorometer using the Qubit dsDNA HS Assay Kit. DNA integrity was confirmed by electrophoresis on a 1.5% agarose gel.

Library preparation was performed from 100 ng of genomic DNA using the MGIEasy FS DNA Library Prep Set (MGI Tech) following the standard protocol. Adapters were ligated, DNA ends repaired, fragments circularized, and DNA nanoballs (DNBs) synthesized before flow-cell loading. Sequencing was conducted on a DNBSeq-G400 platform (MGI Tech) using the DNBSeq PE150 configuration, generating a total of 3,406,660 paired-end reads (2 × 150 bp), corresponding to approximately 170× coverage for a 6 Mb genome. Sequencing used an FCL flow cell (PE150 configuration, four lanes, ~400 million reads/lane); BB2 was loaded onto lane 2. DNB loading was normalized to 1 pM final pool concentration, with 2.18 ng of PCR product loaded for BB2 (target ~3 million reads, 0.75% of lane yield). The ssDNA library was adjusted to 40 fmol/µL, and 25 µL of the DNB preparation was loaded onto the flow cell. Base calling used the DNBSeq-G400’s standard integrated acquisition/processing software (MGI Tech), generating FASTQ files. Read quality was assessed with FastQC v0.12.1 with default parameters (5), and adapter trimming and filtering were conducted with Fastp v0.23.4 under two stringency levels (Q28 and Q30) (--detect_adapter_for_pe --trim_poly_g --overrepresentation_analysis --trim_poly_x --low_complexity_filter --correction --thread 16 --trim_front1 13 --trim_front2 13 --trim_tail1 0 --trim_tail2 0 --cut_tail --cut_window_size 4 --cut_mean_quality 30 --average_qual 28 | 30). The Q30 data set was retained for subsequent assembly.

*De novo* assembly was performed with SPAdes v3.15.5 (--threads 24—memory 120 –-careful) (6); contigs shorter than 500 bp were excluded; the resulting assembly consisted of 187 contigs, totaling 5,252,178 bp, with an N50 of 78,784 bp. Assembly quality was evaluated using QUAST v5.3.0 with default parameters (7), which also detected full-length rRNA operons. Scaffolding was refined with RagTag v2.1.0 with default parameters using *Brevibacillus borstelensis* CP144922 as reference, allowing separation of chromosomal and potential plasmid fragments. Genome completeness and contamination were estimated with CheckM1 v1.2.5 with default parameters (8), yielding values of >91.02% completeness and 7.66% of contamination.

Genome annotation with Prokaryotic Genome Annotation Pipeline (PGAP) (V6.10 with default National Center for Biotechnology Information [NCBI] parameters, method: best-placed reference protein set; GeneMarkS-2+) (9) identified 5,035 CDSs, 6 rRNAs, 59 tRNAs, 5 ncRNAs, 79 pseudogenes, 2 CRISPR arrays, and a single oriC. The assembly consists of 187 contigs, totaling 5,247,696 bp, with 51.74% GC content, an N50 of 78,784 bp, N90 of 17,420 bp, no gaps (*N* = 0), and 86.3% coding density. Functional annotation (eggNOG-mapper v2.1.9 with default parameters) (10) assigned genes to 25 COG categories dominated by S (25.9%), E (10.7%), and K (10.3%). Genes associated with sporulation, stress response, bacillithiol biosynthesis, siderophore production/transport, β-lactamases, multidrug resistance, and multiple esterases/lipases were also detected, supporting the ecological adaptability of *Brevibacillus borstelensis* BB2.

## Data Availability

The whole-genome shotgun project for *Brevibacillus borstelensis* strain BB2 strain has been deposited in DDBJ/ENA/GenBank under the accession JBUBWW000000000. The version described in this paper is JBUBWW000000000. The associated BioProject and BioSample accession numbers are PRJNA1416467 and SAMN54969686, respectively. The sequence read data are under the accession SRR39417049.
